# Supplementary material for: The Identification of APOBEC3G as a Potential Prognostic Biomarker in Acute Myeloid Leukemia and a Possible Drug Target for Crotonoside
Source: Molecules. 2022 Sep 7;27(18):5804. doi: 10.3390/molecules27185804 (PMC9503540; doi:10.3390/molecules27185804)
Supplement: Supplementary file 1 [file molecules-27-05804-s001.zip › molecules-1824534-supplementary.pdf]

Table S1. 2017 European LeukemiaNet cytogenetic risk stratification of acute myeloid leukemia.

Risk category*	Genetic abnormality
**Favorable**	t(8;21)(q22;q22.1); *RUNX1-RUNX1T1*
	inv(16)(p13.1;q22) or t(16;16)(p13.1;q22); *CBFB-MYH11*
	Mutated *NPM1* without *FLT3-ITD* or with *FLT3-ITD low*
	Biallelic mutated *CEBPA*
**Intermediate**	Mutated *NPM1* and *FLT3-ITD high*
	Wild type *NPM1* without *FLT3-ITD* or with *FLT3-ITD low* (without adverse-risk genetic lesions)
	t(9;11)(p21.3;q23.3); *MLLT3-KMT2A*
	Cytogenetic abnormalities not classified as favorable or adverse
**Poor**	t(6;9)(p23;q34.1); *DEK-NUP214*
	t(v;11q23.3); *KMT2A* rearranged
	t(9;22)(q34.1;q11.2); *BCR-ABL1*
	inv(3)(q21.3q26.2) or t(3;3)(q21.3;q26.2); *GATA2,MECOM*(*EVI1*) –5 or del(5q); –7; –17/abn(17p)
	Complex karyotype, monosomal karyotype
	Wild type *NPM1* and *FLT3-ITD high*
	Mutated *RUNX1*
	Mutated *ASXL1*
	Mutated *TP53*
